# Melatonin reshapes the mitochondrial network and promotes intercellular mitochondrial transfer via tunneling nanotubes after ischemic‐like injury in hippocampal HT22 cells

**DOI:** 10.1111/jpi.12747

**Published:** 2021-06-14

**Authors:** Maria Gemma Nasoni, Silvia Carloni, Barbara Canonico, Sabrina Burattini, Erica Cesarini, Stefano Papa, Marica Pagliarini, Patrizia Ambrogini, Walter Balduini, Francesca Luchetti

**Affiliations:** ^1^ Department of Biomolecular Sciences University of Urbino Carlo Bo Urbino Italy

**Keywords:** HT22, melatonin, mitochondrial network, oxygen‐glucose deprivation, tunneling nanotubes

## Abstract

Mitochondrial dysfunction is considered one of the hallmarks of ischemia/reperfusion injury. Mitochondria are plastic organelles that undergo continuous biogenesis, fusion, and fission. They can be transferred between cells through tunneling nanotubes (TNTs), dynamic structures that allow the exchange of proteins, soluble molecules, and organelles. Maintaining mitochondrial dynamics is crucial to cell function and survival. The present study aimed to assess the effects of melatonin on mitochondrial dynamics, TNT formation, and mitochondria transfer in HT22 cells exposed to oxygen/glucose deprivation followed by reoxygenation (OGD/R). The results showed that melatonin treatment during the reoxygenation phase reduced mitochondrial reactive oxygen species (ROS) production, improved cell viability, and increased the expression of PGC1α and SIRT3. Melatonin also preserved the expression of the membrane translocase proteins TOM20 and TIM23, and of the matrix protein HSP60, which are involved in mitochondrial biogenesis. Moreover, it promoted mitochondrial fusion and enhanced the expression of MFN2 and OPA1. Remarkably, melatonin also fostered mitochondrial transfer between injured HT22 cells through TNT connections. These results provide new insights into the effect of melatonin on mitochondrial network reshaping and cell survival. Fostering TNTs formation represents a novel mechanism mediating the protective effect of melatonin in ischemia/reperfusion injury.

## 1 INTRODUCTION

Cerebral hypoxia/ischemia is one of the leading causes of death and long‐term disability in the world. Ischemia is characterized by the disruption of the oxygen/glucose delivery supporting the oxidative metabolism of cells. Furthermore, reperfusion after ischemia compounds brain injury by triggering oxidative stress exacerbating in turn necrotic and apoptotic cell death.^1^, ^2^, ^3^ Mitochondria are the prominent cell organelles affected by the hypoxic/ischemic insult. Indeed, using oxygen to provide ATP through the electron transport chain, mitochondria represent the leading site and target of ROS production. Mitochondrial overproduction of ROS during ischemia/reperfusion is considered one of the leading factors that contribute to cell death, particularly in organs where the energy demand is high, including the brain.^4^ Therefore, reduction in ROS production and the scavenging of mitochondrial ROS aid in the preservation of mitochondrial functions and biogenesis after ischemia/reperfusion injury and are crucial to maintaining and sustaining cell activity and viability.

Mitochondria are extremely dynamic organelles continuously changing their shape, size, and location within cells. Mitochondrial dynamics represent a critical component of cell quality control and are managed through different mechanisms, including biogenesis, fission, and fusion.^5^ Fission and fusion are critical mechanisms in repairing damaged mitochondria because they allow the exchange of material between damaged and nondamaged mitochondria via the fusion process or the segregation of damaged components. Unopposed fission causes mitochondrial fragmentation and is generally associated with metabolic dysfunctions. On the other hand, unopposed fusion results in a hyperfused network and serves to counteract metabolic insults, preserve cellular integrity and protect against mitophagy. Fission and fusion are active processes that require many specialized proteins. Dynamin‐related protein 1 (DRP1), Fis1, and Endophilin B1 are required for mitochondrial fission in mammals, whereas Mitofusin 1 (MFN1), Mitofusin 2 (MFN2), and Optic atrophy 1 (OPA1) are responsible for fusion. Proteins involved in the fission and fusion machinery have been implicated in the mitochondrial shape changes induced by ischemic insults.^6^ Mitochondria can also be transferred between cells.^7^ Mitochondrial transfer represents another important endogenous mechanism in the context of tissue regeneration and stroke because it may support the exogenous replacement of damaged mitochondria, improving cell survival.^8^, ^9^, ^10^ The molecular mechanism(s) through which cells containing dysfunctional mitochondria acquire functional mitochondria from other cells and how this process is regulated remain unclear. Tunneling nanotubes (TNTs) are currently considered as the primary cellular structures mediating cell‐to‐cell mitochondrial transfer. TNTs are dynamic actin‐containing membranous protrusions that connect cells with a small diameter (20‐500 nm) and length of up to 100 μm.^11^ Through TNTs, cells transfer not only mitochondria but also membrane proteins, soluble molecules, and other organelles.^11^ Studies have shown the involvement of TNTs in mitochondrial transport, repair of cell damage, activation of immune responses, and cell metabolic reprogramming.^12^, ^13^, ^14^, ^15^, ^16^

The present study aimed to assess the effect of melatonin during the reperfusion phase on mitochondrial ROS production, dynamics, and TNT formation in HT22 cells exposed to oxygen/glucose deprivation (OGD). Melatonin (N‐acetyl‐5‐methoxytryptamine) is an evolutionarily conserved ubiquitous molecule that has been widely recognized as a broad‐spectrum antioxidant and a potent free radical scavenger.^17^ Melatonin neutralizes ROS^17^, ^18^ and upregulates antioxidant enzymes and downregulates pro‐oxidant enzymes.^19^, ^20^ It also chelates metal ions^21^ and prevents electron leakage from the electron transport chain.^22^ Moreover, melatonin also plays a key role in the regulation of mitochondrial biogenesis by activating Sirtuin 1 (SIRT1) and 3 (SIRT3)—and the peroxisome proliferator‐activated receptor gamma coactivator 1‐alpha (PGC1α).^23^, ^24^, ^25^ Sirtuins are a family of highly conserved NAD+‐dependent deacetylases that act as cellular sensors to detect energy availability and modulate various metabolic processes by activating important signaling pathways. SIRT1 and SIRT3 are known to coordinate cellular energy stores and ultimately maintain cellular energy homeostasis by deacetylating various proteins that induce catabolic processes while inhibiting the anabolic processes.^23^ SIRT3, in particular, resides primarily in mitochondria^26^ and seems to play an important role during ischemic injury. Its overexpression has been shown to protect cortical neurons against oxidative stress by regulating mitochondrial Ca^2^ ^+^ and mitochondrial biogenesis via PGC1α.^27^ Recent data show that melatonin and SIRT3 post‐translationally collaborate in regulating free radical generation and removal from mitochondria.^28^

We report here that melatonin reduces ROS production and improves mitochondrial dynamics and the intercellular transfer of mitochondria via TNTs after OGD followed by reoxygenation (OGD//R). Mitochondria transfer through TNTs may represent an important novel mechanism mediating cell survival after melatonin treatment.

## 2 MATERIALS AND METHODS

### 2.1 Cell culture

Murine hippocampal HT22 cells were generously provided by Professor Herrera Federico, University of Oviedo, Spain. Cells were cultured in DMEM‐HAM'S F12, supplemented with 10% fetal calf serum, l‐glutamine (100 mmol/L), and 1% antibiotics (penicillin, streptomycin). The cells were incubated in a humidified 5% CO_2_ atmosphere at 37℃. At 80% confluence, cells were detached with trypsin‐EDTA, washed and sub‐cultivated in new 25 cm^2^ flasks for 1‐2 days before the experiments.

### 2.2 Simulation of in vitro ischemia with OGD/R and cell treatments

Hypoxia ischemia was simulated by inducing transient oxygen‐glucose deprivation followed by reoxygenation (OGD/R) as previously described.^29^ Briefly, the cells were seeded at a density of 1 × 10^5^ cell/ml and incubated for 24 hours to allow them to adhere. The HT22 cells were maintained in the glucose‐free culture medium and then transferred into a temperature‐controlled (37℃) anaerobic chamber (Billups‐Rothenberg Modular Incubator chamber) containing a gas mixture composed of 5% CO_2_ and 95% N_2_. They were kept in the chamber for 8 hours. Subsequently, the medium was replaced with normal DMEM containing glucose and the HT22 were returned to a normoxic condition for 18 hours of reoxygenation under 5% CO_2_/95% air. Controls were incubated with normal DMEM containing glucose in a humidified incubator with 5% CO_2_/95% air at 37℃ for the same times as the OGD/R cultures. Melatonin (50 μmol/L) was added to the medium immediately after the OGD procedure and maintained at 37℃ for 18 hours (R). This dose of the drug was used based on previous experiments that showed protective effects of melatonin in HT22 serum‐deprived cells^30^ and in rat hippocampal organotypic slice cultures in OGD condition.^31^

### 2.3 Trypan Blue Test

Adherent and floating cells in the culture medium were collected and centrifuged at 300 x g for 5 minutes. Cells were then resuspended in an equivalent volume of 0.4% Trypan Blue solution and counted with a Burker's chamber under light microscopy. Cells excluding Trypan blue were considered viable. The number of viable cells in the control condition was set as 100%.

### 2.4 XXX5‐CFDA assay

Cell viability was measured by 5‐CFDA, AM (5‐Carboxyfluorescein Diacetate, Acetoxymethyl Ester; Thermo Fisher Scientific) assay. 5‐CFDA is a cell‐permeant esterase substrate that measures enzymatic activity and cell membrane integrity, which is needed for intracellular retention of the fluorescent product. Following OGD/R treatment, HT22 cells were incubated with 1 μmol/L 5‐ CFDA in PBS at 37℃ for 30 minutes. Cells were analyzed by FACSCanto II flow cytometry, collecting at least 10 000 events for each sample.

### 2.5 Determination of mitochondrial oxidation by MitoSOX

MitoSOX^TM^ (Thermo Fisher Scientific, Waltham, MA, USA) Red Reagent penetrates into living cells and produces red fluorescence under oxidative damage of mitochondria. Cells collected after treatment were washed and incubated with 5 μmol/L MitoSOX in PBS at 37℃ for 30 minutes. The mean fluorescence intensity (MFI) was produced by FACSCanto II flow cytometry, collecting at least 10 000 cell events for each sample.

### 2.6 Detection of mitochondria by MitoTracker Deep Red (MTDR) staining

Cells were plated on MatTek glass bottom chambers (MatTek Corporation) at a density of 1 × 10^5^ cells/well. Following treatments, the cells were stained with MitoTracker Deep Red (MTDR; Molecular Probes) for the determination and localization of mitochondria in live and fixed cells. For all experimental conditions, cells were incubated with 200 nmol/L MTDR at 37℃ for 15 minutes. After incubation, the cells were examined under a Leica TCS SP5 II confocal microscope (Leica Microsystem, Germany). The images were processed and analyzed using the NIH‐Image J software (National Institutes of Health, Bethesda, MD) and the Form Factor (FF) calculated from the Area (Am) and Perimeter (Pm) using the following formula: FF = Pm^2^/4πAm. FF has the minimal value of 1 when the measured organelle is drawn as a perfect circle. FF values >1 indicate progressive increased mitochondria length and shape complexity.^32^

### 2.7 Mitofusin 2 (MFN2) immunofluorescence analysis

The HT22 cells were grown on 35 mm MatTek glass bottom dishes (MatTek Corporation; density, 1 × 10^5^ cells/well). After treatment, the cells were washed (2X) with PBS and fixed for 15 minutes with 4% paraformaldehyde (pH 7.4) at room temperature. The cells were washed again (2X) with PBS and then permeabilized for 15 minutes with 0.1% Triton X‐100 in PBS at room temperature. Next, the cells were washed (2X) with PBS and incubated for 60 minutes with blocking solution (PBS containing BSA 2%). The cells were then incubated overnight at 4℃ with a monoclonal anti‐mitofusin‐2 antibody (1:50, D2D10, #9482 Cell Signaling) diluted in the blocking solution (PBS containing BSA 0.1%). After being washed (3X) with PBS, the cultures were incubated for 60 minutes with conjugated anti‐rabbit secondary antibody (1:100). Subsequently, the cells were washed (3X) with PBS and fluorescent images were captured by confocal microscopy (Leica Microsystem, Germany). Images were analyzed using NIH‐Image J software (National Institutes of Health, Bethesda, MD).

### 2.8 Protein extraction and immunoblotting

The cells were incubated on ice for 1 hour with a lysis buffer (50 mmol/L Tris, 5 mmol/L ethylenediaminetetraacetic acid (EDTA), 150 mmol/L NaCl, 0.5% Nonidet P‐40, 1 mmol/L phenylmethylsulphonyl fluoride, 1 mmol/L sodium vanadate, and 1 mmol/L sodium fluoride, pH 7.4) containing a protease inhibitor cocktail (Roche Applied Science) then lysed with a Sonicator Ultrasonic Liquid Processor XL (Heat System‐Ultrasonics) and centrifuged at 21 500 g for 10 minutes at 4℃ to remove detergent‐insoluble material. Supernatants were assayed for protein concentration using the Bradford reagent (Sigma‐Aldrich). Total protein extracts (15 μg) were separated by SDS‐PAGE and transferred to PVDF membranes (Thermo Scientific). A ColorBurst™ electrophoresis marker (3 μL/gel, Sigma‐Aldrich) was used for qualitative molecular mass determinations and for visual confirmation of blot transfer efficiency. The blots were then blocked with non‐fat dry milk in TBS‐T (10 mmol/L Tris, 150 mmol/L NaCl, pH 7.6, plus 0.1% Tween‐20) and probed overnight at 4℃ with the following primary antibodies: anti‐SIRT3 (1:1000, polyclonal; Cell Signaling Technology, #2627), anti‐PGC1α (1:1000, polyclonal; Cell Signaling Technology, #2178), anti‐HSP60 (1:1000, monoclonal; Santa Cruz Biotechnology, sc‐13115), anti‐TOM20 (1:1000, monoclonal; Santa Cruz Biotechnology, sc‐17764), anti‐TIM23 (1:1000, monoclonal; Santa Cruz Biotechnology, sc‐514463), anti‐mitofusin‐2 (1:1000, polyclonal; Cell Signaling Technology, #9482), anti‐DRP1 (1:1000, polyclonal; Santa Cruz Biotechnology, sc‐32898), and anti‐OPA1 (1:1000, monoclonal; Santa Cruz Biotechnology, sc‐393296). Membranes were washed in TBS‐T and incubated for 60 minutes with the appropriate secondary antibody diluted 1:4000 (Santa Cruz Biotechnology), followed by washing in TBS‐T. Proteins were visualized by ECL according to the manufacturer instructions (RPN2209, Sigma‐Aldrich). A primary mouse monoclonal antibody against COX IV (1:500, Abcam, mAbcam33985) was used as loading control and for data normalization. Densitometric analyses were performed using the NIH‐Image J software.

### 2.9 Assessment and quantification of tunneling nanotubes (TNTs) and mitochondrial transfer

TNTs were identified as previously described.^33^ Cells were washed with PBS, fixed for 5 minutes in 3.7% formaldehyde, and permeabilized with 0.1% triton X‐100 for 10 minutes. The cells were then stained for F‐actin by incubation with Phalloidin‐FITC labeled (P5282, Sigma‐Aldrich) (300 nmol/L) for 40 minutes at RT. Finally, the cells were observed and analyzed by Leica TCS‐SL confocal microscope equipped with Argon and He/Ne laser sources. Single plane confocal images (optical thickness 0.8 µm) were taken at 63× magnification. TNTs length and size were evaluated with the LAS‐AF SP5 software (Leica Microsystems). The number of TNTs was quantified in 10 fields randomly selected using the Cell Counter plugin for the NIH‐Image J software and represented as average ± SD.^34^ The number of TNTs with inside mitochondria was calculated in double‐labeled cells analyzing at least 20 images for each experimental group. The results are reported as a percentage of TNTs with mitochondria in relation to a total number of TNTs counted in each experimental condition. Data were collected from three separated experiments. The transfer of mitochondria was evaluated according to Abounit et al.^35^ Briefly, HT22 cells labeled with MTDR (donor cells) and HT22 unlabeled cells (acceptor cells) were mixed in a 1:1 ratio. Cells were then assessed by flow cytometry (FACSCanto II flow cytometer) and the presence of MTDR in unlabeled‐HT22 cells taken as index of mitochondria transfer among cells.

### 2.10 Transmission Electron Microscopy (TEM)

The HT22 cells were seeded in 75 cm^2^ flasks at a density of 2 × 10^6^ cells/well and left to adhere for 24 hours. After treatments, the cells were washed, prefixed in situ with 2.5% glutaraldehyde in 0.1 mol/L phosphate buffer for 15 minutes, gently scraped and centrifuged at 300 x *g* for 10 min. The pellets were fixed in 2.5% glutaraldehyde for an additional 45 minutes at room temperature. After two washes in 0.1 mol/L phosphate buffer, the cells were postfixed for 1 hour in 1% OsO_4_, dehydrated in a graded series of increasing concentrations of ethanol and embedded in araldite. Thin sections were counterstained with uranyl acetate and lead citrate. Ultrastructural analysis was performed with a transmission electron microscope (Philips) and imaged with an SIS MegaView III camera (Soft Imaging System).^36^ Mitochondria were analyzed for major/minor axis using the NIH‐Image J software plugin for mitochondrial morphology. The parameters obtained from these analyses were then used to calculate the Aspect Ratio (AR), that is, the ratio between the major and minor axes of the ellipse equivalent to the mitochondrion. The final values are indicative of mitochondrial elongation and fragmentation.^37^

### 2.11 Statistical analyses

Quantitative data are expressed as mean ± SD on the basis of at least three independent experiments. Differences between groups were analyzed using a one‐way analysis of variance (one‐way ANOVA), followed by Newman‐Keuls Multiple Comparison and Tukey post hoc test. A *P*‐value ˂.05 was considered significant. All statistical analyses were performed using GraphPad Prism 5.0 (GraphPad software).

## 3 RESULTS

### 3.1 Melatonin preserves cell viability after OGD/R in HT22 cells

**FIGURE 1:**
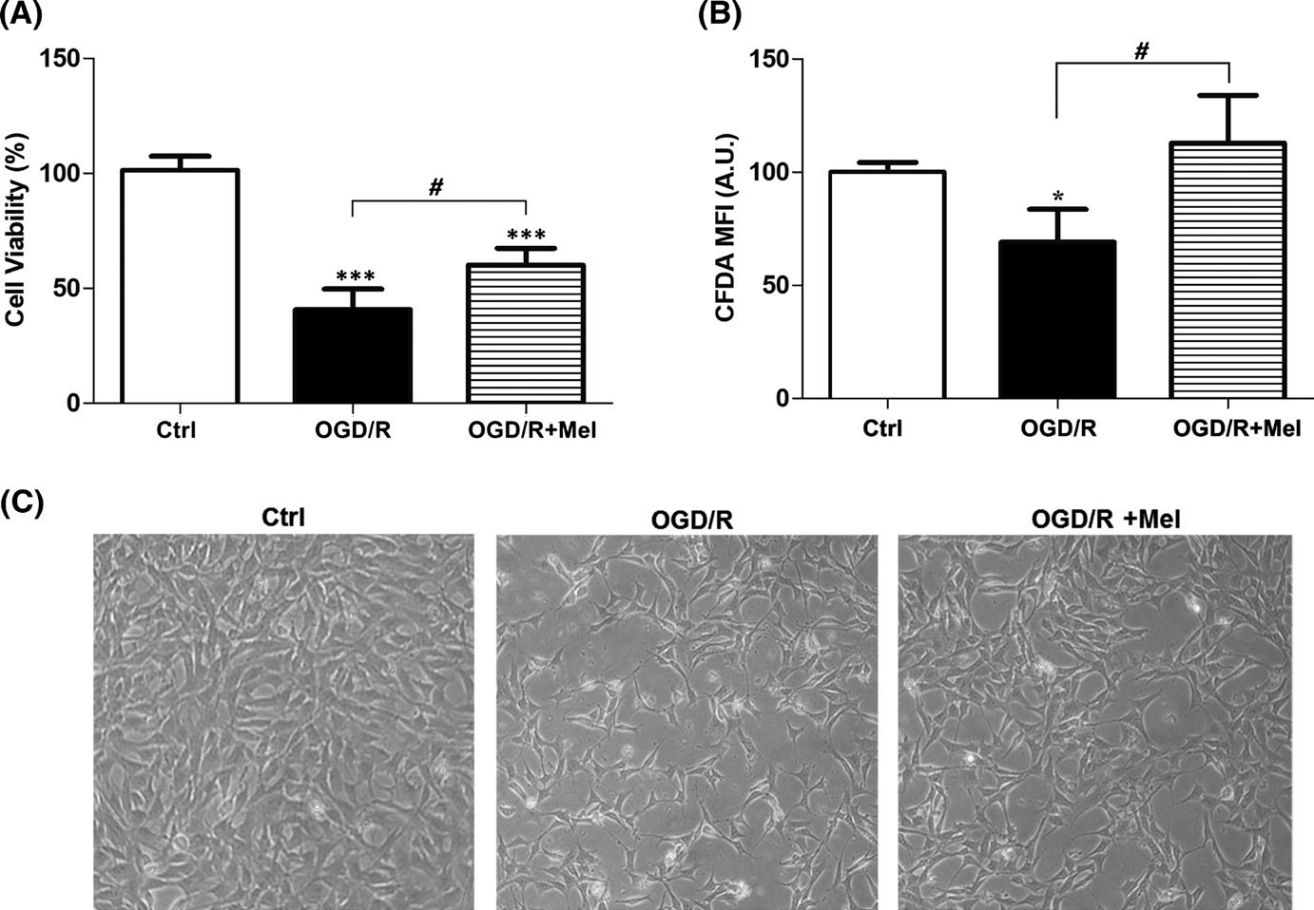
Effect of melatonin on HT22 cell viability after OGD/R treatment. A, the percentage of viable cells analyzed by the Trypan Blue exclusion test in untreated cells (Ctrl), 8 h OGD‐exposed cells followed by 18 h reoxygenation (OGD/R) and 8 h OGD‐exposed cells followed by 18 h reoxygenation in the presence of 50 µmol/L melatonin (OGD/R+Mel). B, 5‐CFDA mean fluorescence intensity (MFI) in Ctrl, OGD/R, and OGD/R+Mel cells. Mean values were converted to arbitrary units (AU) setting control as 100. C, morphological changes in Ctrl, OGD/R, and OGD/R+Mel cells acquired by an optical microscope with 10× objective. Each value is expressed as percentage ±SD (N = 3 independent experiments performed in triplicate); *P < .05, ***P < .001 vs Ctrl; # *P* < .05 vs OGD/R+Mel

The protective effect of melatonin in HT22 cells after OGD/R was assessed by measuring cell viability using both the Trypan Blue and the 5‐CFDA assays. As shown in Figure 1, cells exposed to 8 hours OGD followed by 18 hours of reoxygenation had significantly lower viability (60% reduction compared to the control condition; Figure 1A, *P* < .001). Treatment with 50 μmol/L melatonin during the reoxygenation phase significantly improved cell viability compared to the OGD/R condition (Figure 1A, *P* < .05). The 5‐CFDA assay, an indicator of cell enzymatic activity and viability, also showed a significant decrease in the OGD/R condition. This effect was reversed by melatonin (Figure 1B, *P* < .05 OGD/R+Mel vs OGD/R). The representative bright‐field microscopy images reported in Figure 1C show that the OGD/R cells lost their original flat, elongated morphological features. By contrast, melatonin‐treated cells showed morphological features similar to control cells, with reduced floating cells and increased flat cells.

### 3.2 Melatonin reduces mitochondrial ROS production

**FIGURE 2:**
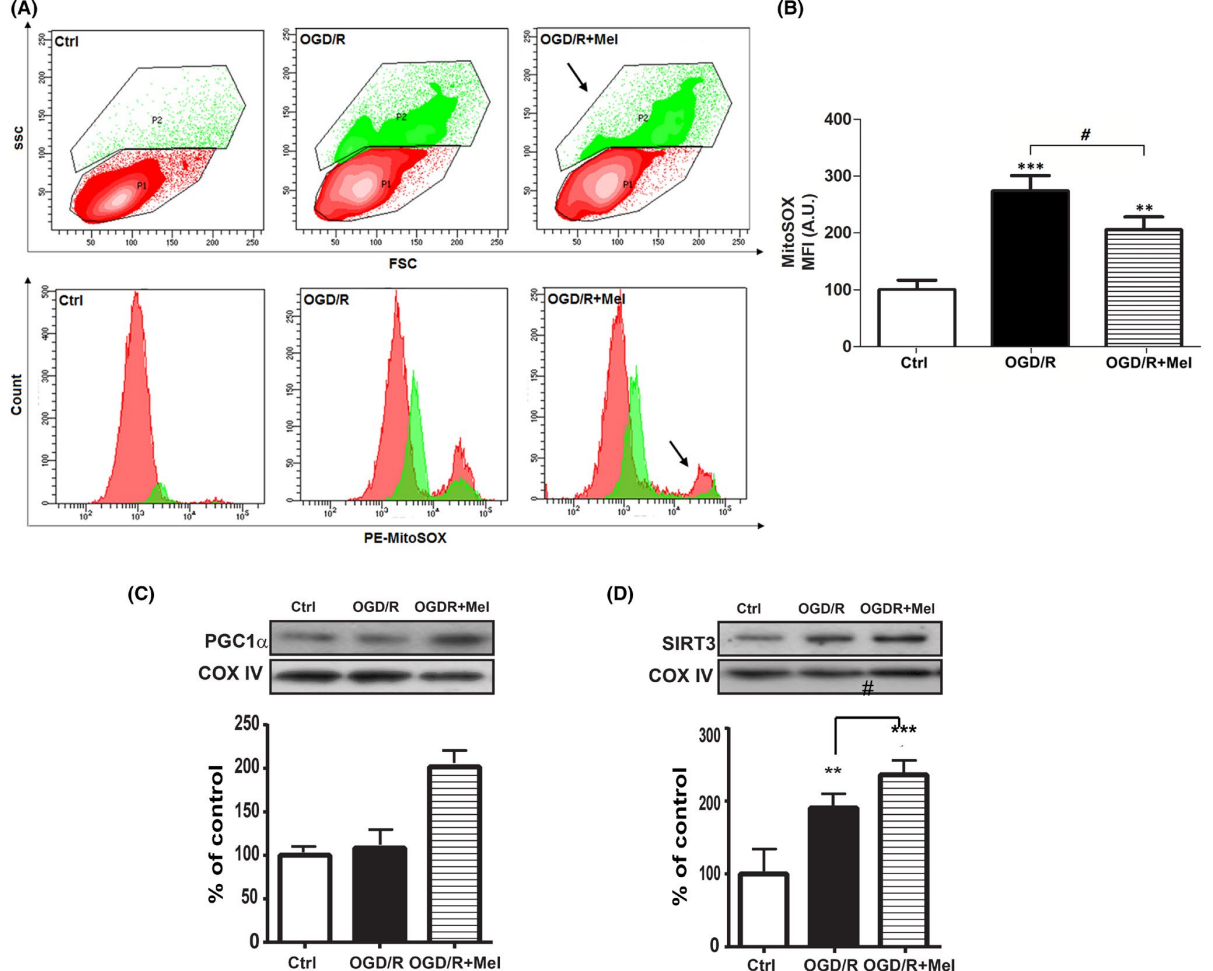
Effect of melatonin on mitochondrial ROS production and PGC1α and SIRT3 expression in OGD/R‐injured HT22 cells. A, representative flow cytometric contour plots (upper panels) and histograms (lower panels) of MitoSox Red labeling in untreated cells (Ctrl), 8 h OGD‐exposed cells followed by 18 h reoxygenation (OGD/R), and 8 h OGD‐exposed cells followed by 18 h 50 µmol/L melatonin reoxygenation (OGD/R+Mel). P1 (red area) and P2 (green area) indicate cells with low and high MitoSOX MFI, respectively. Arrows indicate the MFI decrease of the P2 population. B, flow cytometry analysis of MitoSOX Red labeling in Ctrl, OGD‐R, and OGD/R+Mel. Results are expressed as mean fluorescence intensity (MFI) converted to arbitrary units (AU) setting control as 100. Each value is expressed as a relative mean ± SD (N = 3 independent experiments performed in triplicate); **P < .05, ***P < .001 vs Ctrl; *#* *P* <.05 vs OGD/R+Mel. Representative Western blots and quantitative evaluation of PGC1α (C) and SIRT3 (D) expression in Ctrl, OGD/R, and OGD/R+Mel cells. Data normalized to the loading control COX IV are expressed as % of control and are the mean ±SD (N = 3 independent experiments); **P < .01; ***P < .001 vs Ctrl, #*P* < .05, ###*P* < .001 vs OGD/R+Mel

To assess ROS production, we labeled the HT22 cells with MitoSOX, a fluorogenic dye for highly selective mitochondrial superoxide detection in live cells, analyzing samples by flow cytometry. As shown in Figure 2A, in our experimental conditions the flow cytometric analysis of the MitoSox labeling revealed two cell populations, that is, MitoSOX low‐positive cells (P1, red area) that identified the cell subset with a lower amount of ROS, and MitoSOX high‐positive cells (P2, green area) that identified the cell subset with a higher amount of ROS. The histograms represent the MFI of the P1 and P2 population. The results show increased mitochondrial ROS in OGD/R condition if compared to Ctrl (Figure 2A, lower panel). After melatonin, we observed a decrease in MitoSOX positive cells (Figure 2A, arrows) compared to the OGD/R condition. Figure 2B shows the quantification of the MFI collected in the three experimental conditions where clearly appears the significant increase of mitochondrial ROS production in OGD/R compared to the control condition (~2.7‐fold, *P* < .001; Figure 2B) and its reduction after reoxygenation in the presence of melatonin (*P* < .05; Figure 2B).

We also assessed PGC1α and SIRT3, which are implicated in neuroprotection under oxidative stress, acting synergistically to maintain active ROS defense and mitochondrial biogenesis.^38^ OGD/R did not affect PGC1α expression (Figure 2C), in keeping with previous findings,^39^ but significantly increased the expression of SIRT3 (Figure 2D, *P* ≤ .01). Treatment with melatonin significantly increased the expression of PGC1α compared to both the control and the OGD/R conditions (Figure 2C, *P* ≤ .001). In addition, melatonin increased SIRT3 expression above the OGD/R condition (Figure 2D, *P* < .05).

### 3.3 Melatonin preserved mitochondrial mass affected by OGD/R

**FIGURE 3:**
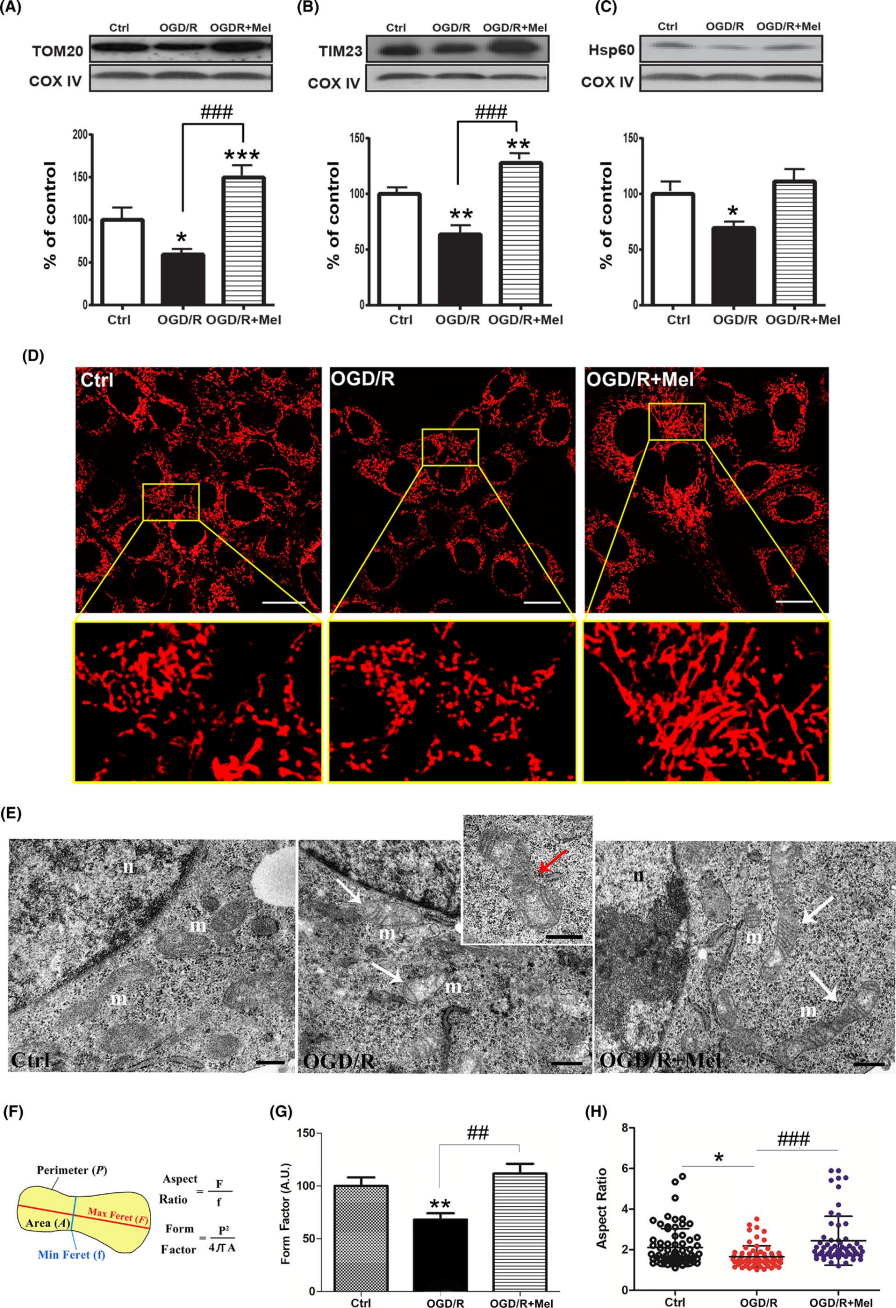
Effect of melatonin on mitochondrial mass in OGD/R‐injured HT22 cells. Representative Western blots and quantitative evaluation of TOM20 (A), TIM23 (B), and Hsp60 (C) expression in untreated HT22 cells (Ctrl), 8 h OGD‐exposed cells followed by 18 h reoxygenation (OGD/R), and 8 h OGD‐exposed cells followed by 18 h 50 µmol/L melatonin reoxygenation (OGD/R+Mel). Data normalized to the loading control COX IV are expressed as % of control and are the mean ± SD (N = 3 independent experiments); *P < .05, **P < .01, ***P <.001 vs Ctrl; *###P* < .001 vs OGD+Mel. D, representative confocal images of mitochondrial morphology in Ctrl, OGD/R, and OGD/R+Mel cells. Cells were stained with 100 nmol/L MitoTracker Deep Red (MTDR). The region bounded by box is shown as an enlarged region for each experimental condition. Scale bars: 20 μm. E, TEM analysis of mitochondrial structure in Ctrl, OGD/R, and OGD/R+Mel cells. Mitochondria (*m*) appear rounded with irregular and disorganized cristae in OGD/R cells (white arrows). The insert shows mitochondria with the morphologic constriction point (red arrow). On the contrary, tubular elongated mitochondria with straight and parallel cristae are visible in OGD/R+Mel cells (white arrows). *n,* Nucleus; Scale bar: 0.5 μm. F, graphical representation of morphological meaning of the Form Factor (FF) and the Aspect Ratio (AR) parameters. G, FF quantification in Ctrl, OGD/R, and OGD/R+Mel cells. FF values were converted to arbitrary units (AU) and expressed as % of Ctrl (10 images were assessed for each experimental condition); H, AR quantification in Ctrl, OGD/R, and OGD/R+Mel cells. Scatter plot represents the AR values distribution. Numbered data points represent individual mitochondria manually traced. 20 images were analyzed and at least n = 50 mitochondria were quantified for each experimental group. Values were expressed as mean ± SD (N = 3 independent experiments performed in triplicate); *P < .05, **P < .01 vs Ctrl, *##P* < .01, *###P* < .001

To assess the regulation of mitochondrial mass and biogenesis, which is crucial to preserving cell viability, we assessed the expression of the mitochondrial import receptor subunits TOM20 and TIM23, and of the matrix protein HSP60, as indicators for mitochondrial mass. OGD/R significantly reduced the expression of TOM20 and TIM23 (Figure 3A, *P* ≤.05; Figure 3B, *P* ≤ .01), and also reduced the expression of the matrix protein HSP60 (Figure 3C, *P* ≤ .05). Melatonin completely reversed the OGD/R‐induced loss of these proteins. Indeed, after melatonin treatment, the expression of both TOM20 and TIM23 was higher than it was in the control condition (Figure 3A and B, *P* ≤ .001). To better characterize mitochondria preservation, we analyzed their morphology by confocal microscopy and TEM. After OGD/R, MTDR labeling showed a clear reduction in mitochondrial mass, which appeared smaller and fragmented compared to the control condition (Figure 3D). After melatonin treatment, the mitochondrial mass appeared elongated showing a tubular morphology similar to that of the control condition (Figure 3D). TEM analysis showed that, unlike in the control condition, after OGD/R the mitochondria cristae were irregular, dilated, and without a parallel distribution. In addition, the mitochondria appeared round with morphologic constriction points (Figure 3E, insert). By contrast, after melatonin treatment, the mitochondria conserved elongated tubular shapes and straight, parallel cristae, comparable to the control condition (Figure 3E). Morphological changes were quantified in both confocal microscopy and TEM measuring the Form Factor (FF) parameter, reflecting the complexity and branching aspects of mitochondria (Figure 3F and G) and the Aspect Ratio (AR), an indicator of mitochondria length (Figure 3F and 3H). After OGD/R, there was a significant reduction in the FF compared to Ctrl (Figure 3G), indicating fragmented and discontinuous mitochondria. Treatment with melatonin improved the FF parameter (Figure 3G; *P* ≤ .01 vs OGD/R), indicating a better‐preserved mitochondria morphology. A more detailed image analysis was performed in TEM images on single mitochondria. The AR values showed that the mitochondria length was significantly lower in OGD/R cells compared to healthy Ctrl cells (Figure 3H; *P* ≤ .05). Melatonin treatment significantly increased the AR value (Figure 3H; *P* ≤ .001 vs OGD/R), showing elongation of the mitochondria structure.

### 3.4 Melatonin restores mitochondrial fusion/fission dynamics affected by OGD/R

**FIGURE 4:**
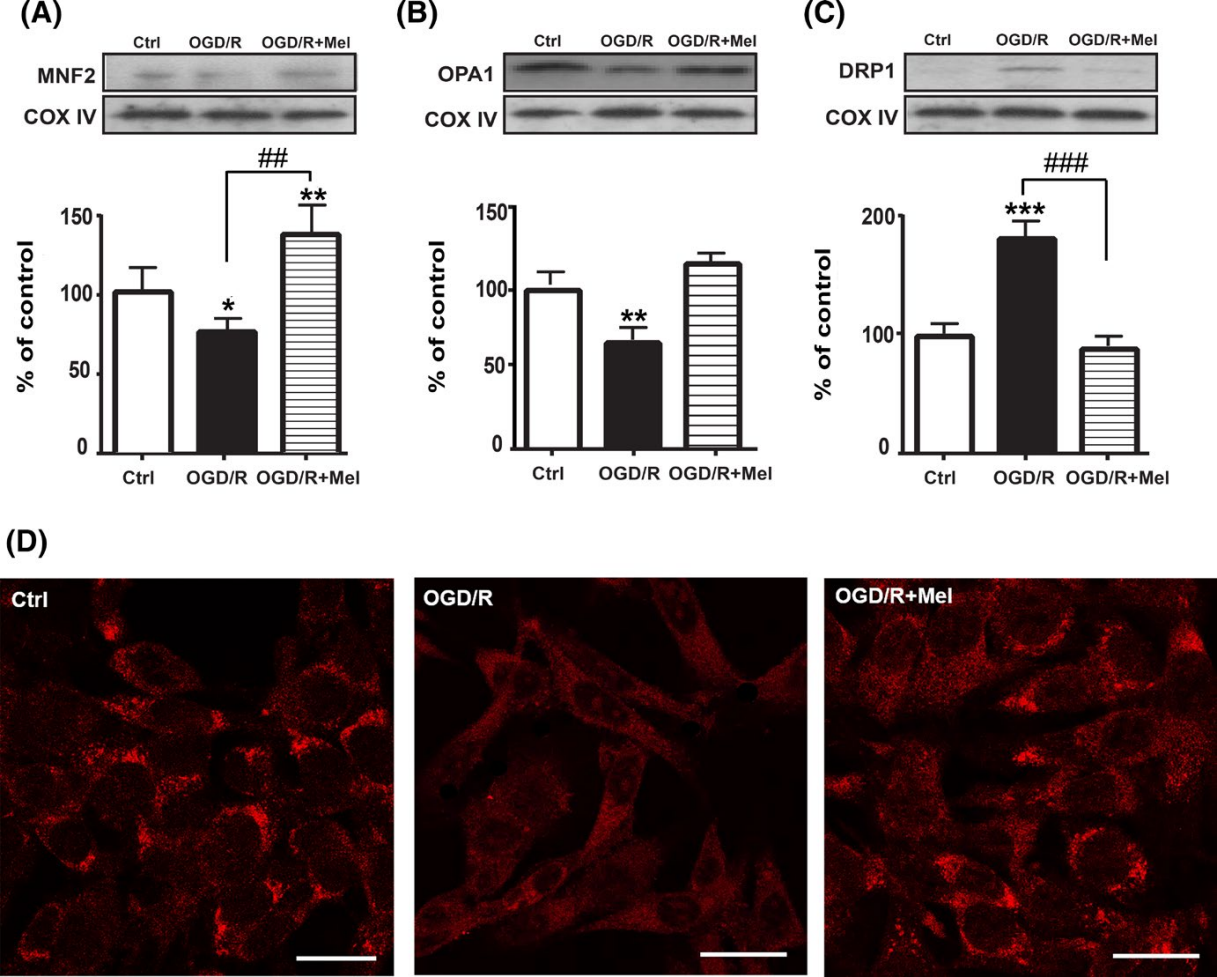
Effect of melatonin on the mitochondrial fusion/fission proteins. Representative Western blots and quantitative evaluation of MFN2 (A), OPA1 (B), and DRP1 (C) expression in untreated cells (Ctrl), 8 h OGD‐exposed cells followed by 18 h reoxygenation (OGD/R), and 8 h OGD‐exposed cells followed by 18 h 50 µmol/L melatonin reoxygenation (OGD/R+Mel). Data normalized to the loading control COX IV are expressed as % of control and are the mean ± SD (N = 3 independent experiments performed in triplicate); *P < .05; **P < .01; ***P < .001 vs Ctrl; ##*P* < .01, ###*P* < .001 vs OGD+Mel. D, confocal images of intracellular localization of MFN2 by immunofluorescence in Ctrl, OGD/R, and OGD/R+Mel cells. Scale bars: 25 µm

Since the dynamic balance between the fusion and fission of mitochondria determines their morphology and allows their immediate adaptation to stress conditions,^40^ we also analyzed proteins involved in the mitochondrial fusion/fission dynamics. OGD/R injury significantly reduced the expression of the mitochondrial fusion‐related factors MFN2 (Figure 4A, *P* < .05) and OPA1 (Figure 4B, *P* < .01). Conversely, the mitochondrial fission factor DRP1 was significantly upregulated after OGD/R (Figure 4C, *P* < .001). Melatonin restored the levels of mitochondrial fusion‐related proteins (Figure 4A and B) and reported the expression of DRP1 to the control levels (Figure 4C). MFN2 was also examined by confocal immunofluorescence, confirming that melatonin‐treated cells had a higher level of labeling compared to OGD/R cells, as revealed by the cytoplasmic punctate labeling of the protein (Figure 4D).

### 3.5 Melatonin induces mitochondria transfer via tunneling nanotubes (TNTs)

**FIGURE 5:**
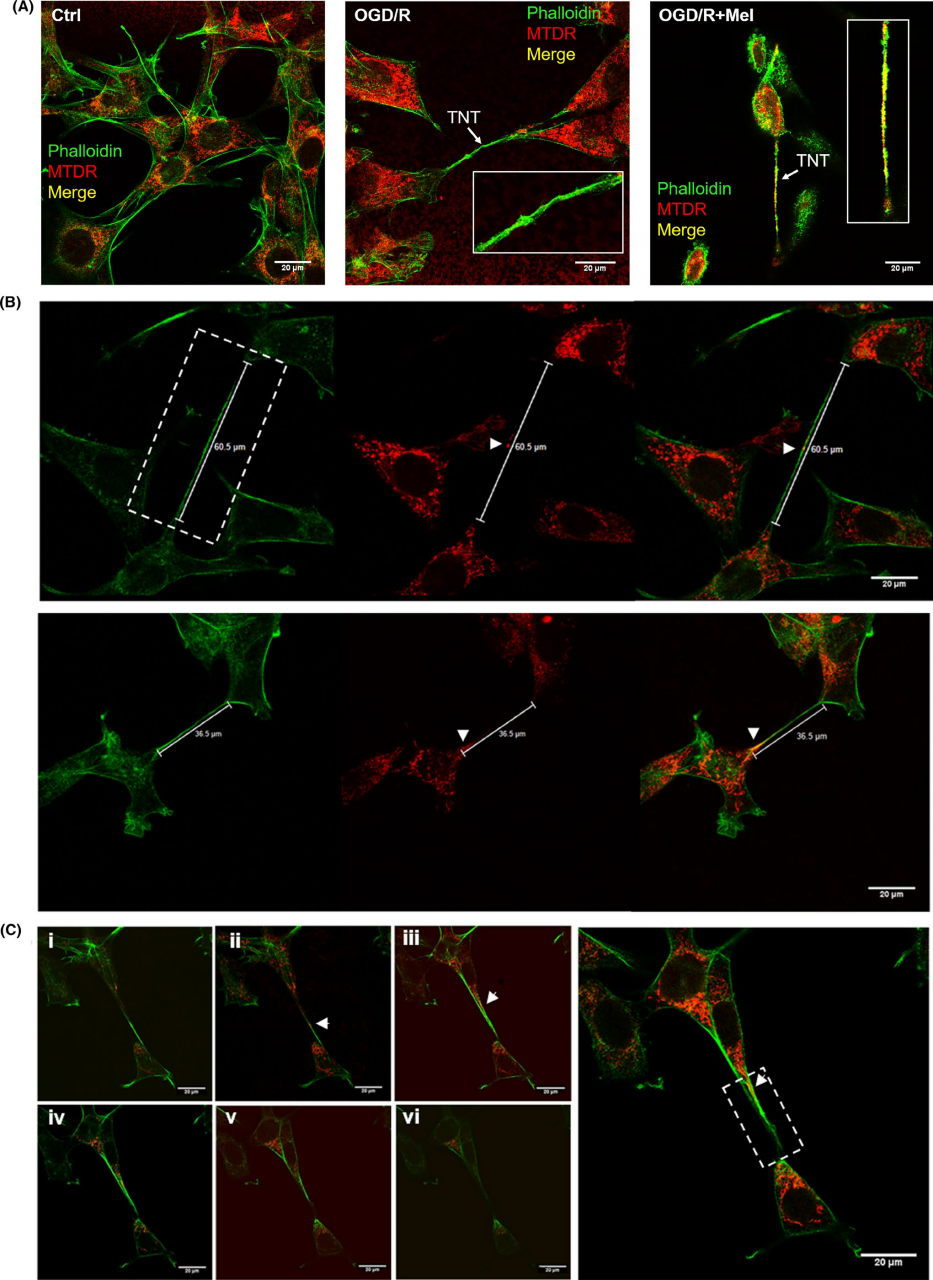
Effect of melatonin on OGD/R‐induced TNTs formation and mitochondrial transfer in HT22 cells. A, confocal images of co‐staining with phalloidin (green) and MitoTracker Deep Red (MTDR, red) of untreated cells (Ctrl), 8 h OGD‐exposed cells followed by 18 h reoxygenation (OGD/R) and 8 h OGD‐exposed cells followed by 18 h 50 µmol/L melatonin reoxygenation (OGD/R+Mel). Arrows indicate tunneling nanotubes (TNTs). The overlay (right panel) shows the co‐localization (yellow) of phalloidin (green) and MTDR (red) indicating the presence of mitochondria within TNTs in OGD/R+Mel cells. B, Characterization of TNTs size in OGD/R+Mel co‐labeled with MTDR (red) and phalloidin (green) indicating the mitochondrial transfer (yellow, white arrows). C, z‐stack images (i‐vi) show mitochondria inside TNT structures (yellow, arrows). Z‐stack acquisitions (*x*, *y*, *z*) were performed through confocal microscopy (TCS SP5 X, Leica Microsystems). Scale bars: 20 µm

**FIGURE 6:**
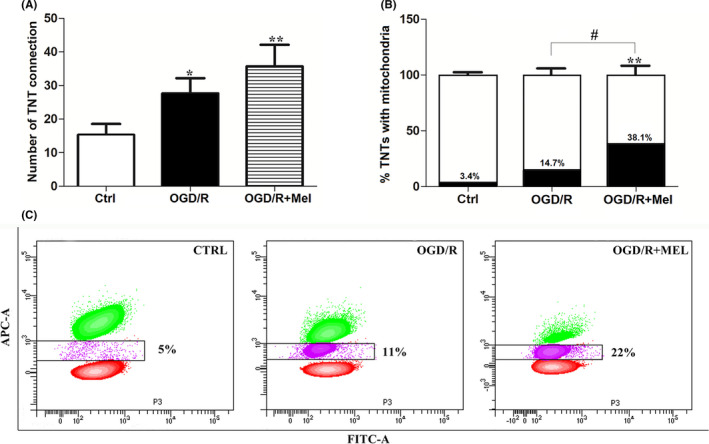
TNTs quantification and mitochondrial transfer in OGD/R+Mel HT22 cells. A, counting of TNTs in untreated cells (Ctrl), 8 h OGD‐exposed cells followed by 18 h reoxygenation (OGD/R) and 8 h OGD‐exposed cells followed by 18 h 50 µmol/L melatonin reoxygenation (OGD/R+Mel). Cell counting was performed as described in Material and Methods. Data are expressed as the mean ± SD (N = 3 independent experiments performed in triplicate); *P < .05, **P < .01 *vs* Ctrl. B, quantification of TNTs with mitochondria in Ctrl, OGD/R, and OGD/R+Mel cells. The percentage was calculated analyzing at least 20 images and n = 25 TNTs were assessed for each experimental group. The number of TNTs was set up to 100 and relative % of TNT with mitochondria is expressed as the mean ± SD (N = 3 independent experiments performed in triplicate); **P < .01 vs Ctrl; #*P* < .05 vs OGD+Mel. C, representative flow cytometric contour plots of mitochondrial transfer in Ctrl, OGD/R, and OGD/R+Mel cells. HT22 Mitotracker Deep Red‐stained cells (red population) co‐cultured with unstained cells (green population) in a ratio of 1:1. The percentage of mitochondrial transfer has been quantified as the number of unstained cells that took up the Mitotracker Deep Red‐red fluorescence (violet population). The experiment was repeated twice with similar results

The mitochondria intercellular transfer via TNTs has been associated with the recovery of injured cells.^41^ To investigate the presence of mitochondria in TNTs in our experimental conditions, we preliminary assessed three different mitochondrial dyes, that is, MitoTracker Red, TMRE, and Mitotracker Deep Red. All dyes allowed us to detect mitochondria into TNTs (data not shown). However, the Mitotracker Deep Red was well‐retained after aldehyde fixation and allowed better co‐localization experiments with phalloidin (green fluorescence), which labels F‐actin structures.^42^, ^43^ Thus, the latter dye was used to characterize TNTs and mitochondria in all subsequent experiments. Typical actin‐TNT labeled structures in HT22 cells showed an average length and diameter of 30‐60 µm and of 300‐500 nm, respectively (Figure 5Aand B), in agreement with previous data.^11^ Confocal z‐stack images showed a cluster of mitochondria adjacent to the entrance and some mitochondria inside the TNTs, as shown by the yellow co‐localization of red and green fluorescence in TNT structures (Figure 5C). The number of TNTs was low in control conditions (Figure 6A). By contrast, TNTs were found in higher amounts after OGD/R and further increased after exposure to melatonin (Figure 6A). Co‐labeling revealed a higher number of TNTs with mitochondria inside in the OGD/R+Mel (38.1%) compared to the OGD/R (14.7%), and the control condition (3.4%) (Figure 6B). To further assess the increased mitochondrial transfer after melatonin treatment, we used flow cytometric analysis. In these experiments, MTDR‐labeled cells (Figure 6C, red population) were co‐cultured with unstained cells (Figure 6C, green population) in the ratio 1:1. Mitochondrial transfer was quantified as the number of unstained cells that took up the Mitotracker Deep Red‐red fluorescence (violet population) in the three different experimental conditions. The transfer was quantified as 22% in OGD/R+Mel, 11% in OGD/R, and 5% in the Ctrl condition (Figure 6C), in line with the results reported in Figure 6B.

## 4 DISCUSSION

Mitochondrial dysfunction is considered one of the hallmarks of ischemia/reperfusion injury^2^, ^44^; therefore, maintaining mitochondrial function is crucial to neuron survival and neurological improvement. We report here that melatonin reduces ROS formation and increases the expression of mitochondrial fusion proteins repressed by OGD/R in hippocampal HT22 cells, preventing mitochondrial dysfunction. However, the most interesting finding of the present study is the transfer of mitochondria via TNTs induced by melatonin, which may represent an important novel mechanism through which melatonin may support cell survival in ischemic conditions.

Neurons are highly susceptible to oxidative stress due to their high polyunsaturated lipid content, high oxygen consumption, and low concentration of antioxidants and related enzymes.^45^ Accordingly, neurons have stringent quality control mechanisms to ensure a healthy mitochondrial network to promote survival and neurological improvement. Our results show that melatonin reduces the amount of mitochondrial ROS induced by ischemia/reperfusion also thanks to its well‐known scavenging effect on free radicals.^17^, ^46^ In addition to its scavenging of ROS, melatonin also enhances mitochondrial antioxidant enzymes, promotes respiration and ATP production, and reduces activation of the intrinsic apoptosis pathway. Here, we found that melatonin enhances SIRT3 and PGC1α expression. SIRT3 is mainly found in mitochondria, where it mediates, together with SIRT4 and SIRT5, the post‐translational modifications of lysine residues. These are reversible actions regulating proteins involved in a wide variety of molecular pathways, including respiratory enzymes controlling mitochondrial function and ROS generation. SIRT3 was upregulated after OGD/R in the HT22 cells, which is in agreement with what has previously been found in PC12 cells,^39^ and its expression was further increased by melatonin. This result is in line with the finding that overexpression of SIRT3 protects mitochondria against OGD injury.^27^, ^38^ SIRT3 functions as a downstream target gene of PGC1α and mediates the PGC1α effects on cellular ROS production and mitochondrial biogenesis. In our experimental conditions, the expression of PGC1α after OGD/R was not increased, in contrast to what it was found for SIRT3. Although the reason of this discrepancy is not clear, we found conflicting results in the literature regarding PGC1α expression after OGD/R, which was found increased in some experimental models^47^, ^48^ but not in others.^49^, ^50^ Nevertheless, the concomitant increase in SIRT3/PGC1α after melatonin treatment indicates that the SIRT3/PGC1α pathway may be involved in its protective effect, as suggested by previous studies.^38^, ^39^ Our findings also show that melatonin upregulates the expression of mitochondrial fusion‐related factors, such as MFN2 and OPA1. These proteins promote elongated interconnected networks that enable the replenishment of damaged mitochondrial DNA and intracellular energy distribution.^51^, ^52^ Fused mitochondria allow efficient exchange of metabolites, increase ATP production, and decrease ROS production. By contrast, fission causes mitochondrial fragmentation and is generally associated with metabolic dysfunctions. We found that melatonin restored the mitochondrial fusion‐related proteins OPA1 and MFN2 and reduced the fission protein DRP1. Interestingly, downregulation of OPA1 has been associated with both aberrant mitochondrial cristae remodeling and low energy production,^53^ and controlled transgenic overexpression of OPA1 can “tighten” cristae junctions, limiting cytochrome c release and providing protection from ischemic brain damage following stroke.^54^ Furthermore, a causal role of DRP1 mediated fission in apoptosis and cell death following in vitro and in vivo ischemic stroke has also been reported.^55^ In line with this observation, we found that after melatonin treatment, mitochondria conserved elongated tubular shapes and straight and parallel cristae compared to the control condition. Melatonin also preserved the membrane translocases TOM20 and TIM23 and the matrix protein Hsp60, proteins involved in mitochondrial biogenesis,^56^ which, when defective, contributes to mitochondrial dysfunction and neurodegeneration.^57^

An interesting novel finding of the present work is that melatonin, in addition to preserving mitochondria from OGD/R injury, also promotes mitochondrial transfer between cells. A growing body of evidence indicates that the distribution of mitochondria within cells depends on numerous factors, including ATP requirements, cellular repair, and renewal of damaged organelles. Cells can also transfer mitochondria via TNTs. TNTs are dynamic structures that connect cells, and their formation is induced by exposure to several kinds of environmental conditions, such as inflammation, infection, and oxidative stress.^58^, ^59^ Intercellular organelle transfer through these structures could compensate for damaged organelles, resulting in cell recovery.^16^ Mitochondrial trafficking via TNTs appears to be critical to the turnover and maintenance of mitochondrial quality control and would seem to be of particular importance in mesenchymal stem cells (MSCs).^60^, ^61^, ^62^ Examples of mitochondria transfer have been shown between stem/mesenchymal stem cells and different target cells, such as endothelial, pulmonary alveolar epithelial, renal tubular, neuronal, and cancer cells. Liu et al^62^ demonstrated that MSCs could repair postischemic endothelial cells with dysfunctional mitochondria by transferring functional mitochondria from healthy cells through TNT formation, thereby rescuing aerobic respiration and protecting endothelial cells from apoptosis. Recently, several authors demonstrated mitochondria transfer *via* TNTs from multipotent MSCs to neuronal cells after ischemic injury.^60^, ^61^, ^63^, ^64^ Our results showing TNT formation in HT22‐injured cells are consistent with these findings. In OGD/R conditions, the number of TNT connections was lower and with a lower number of mitochondria into TNTs compared to what was found after melatonin treatment. Whether the improved transfer of mitochondria between cells occurring after melatonin treatment could be due to its upstream protective effect on the mitochondrial network and which signaling pathways are involved need to be investigated.

In summary, this study provides new insights into the effect of melatonin on the reshaping of the mitochondrial network and neuronal survival. To the best of our knowledge, this is the first evidence showing that melatonin promotes TNT formation and mitochondria transfer between neuronal cells. We speculate that if this effect also occurs in vivo, melatonin could represent a potential tool to improve survival after MSC cell transplantation in different organs. Experiments are needed to test this hypothesis.

## CONFLICT OF INTEREST

The authors of this paper declare that they do not have any conflict of interest.

## AUTHORS CONTRIBUTION

MG N. and SC conceptualized and designed the study, performed most of the experiments and the acquisition of the data, drafted the initial manuscript, and approved the final manuscript as submitted. BC SB EC MP, and PA performed the experiments and methodology and provided technical help. SP contributed reagents, write assistance, and acquisition of funding. WB and FL designed the study, critically reviewed and revised the manuscript, and approved the final manuscript as submitted. All authors approved the final manuscript as submitted and agree to be accountable for all aspects of the work.
